# The influence of the macro-environment on physical activity: a multilevel analysis of 38 countries worldwide

**DOI:** 10.1186/1479-5868-9-110

**Published:** 2012-09-11

**Authors:** Jizzo R Bosdriesz, Margot I Witvliet, Tommy LS Visscher, Anton E Kunst

**Affiliations:** 1 Department of Public Health, Academic Medical Centre, University of Amsterdam, PO Box 22660, Amsterdam, 1100 DD, the Netherlands; 2 Section Prevention and Public Health, Institute of Health Sciences, VU University, Amsterdam, the Netherlands; 3 Research Centre for the Prevention of Overweight, VU University Amsterdam/Windesheim University of Applied Science, PO Box 10090, Zwolle, 8000 GB, The Netherlands; 4 EMGO Institute for Health and Care Research, VU University Medical Centre, Amsterdam, the Netherlands

**Keywords:** Physical activity, Environment, Multilevel analysis, World Health Survey

## Abstract

**Background:**

As indicated by the ANGELO framework and similar models, various environmental factors influence population levels of physical activity (PA). To date attention has focused on the micro-level environment, while evidence on the macro-level environment remains scarce and mostly limited to high-income countries. This study aims to investigate whether environmental factors at macro-level are associated with PA among a broader range of countries.

**Methods:**

Data from the World Health Survey (WHS) was used to analyze 177,035 adults from 38 (mostly low and middle income) countries. The International Physical Activity Questionnaire-Short Form (IPAQ-S) was used to measure PA. Respondents were classified as active or inactive for vigorous PA, moderate PA and walking. Multilevel logistic regression was performed to assess associations between macro-level environmental factors and the prevalence of PA, with control for individual-level socioeconomic factors.

**Results:**

The prevalence of PA varied widely between countries and types of PA (5.0%-93.8%). A negative association was found between gross domestic product and PA, odds ratios for men were 0.76 (95% CI: 0.65-0.89) for moderate PA and 0.79 (95% CI: 0.63-0.98) for walking. A higher temperature was associated with less PA (all types) and higher urbanization was associated with less vigorous and moderate PA. More gender equality was also associated with more walking for women. Governmental functioning and literacy rate were not found to be associated with any type of PA.

**Conclusions:**

This exploratory study indicates that factors such as climate, economic development and cultural factors are determinants of the level of overall PA at national levels. This underlines the suggestion that the macro-environment should be regarded as an important influence on PA.

## Background

Physical activity (PA) is of vital importance for the prevention and treatment of obesity and poor health in general. Small amounts of light PA can have large protective effects [1,2]. Levels of PA are in part determined by individual characteristics, as well as by environmental factors [3,4]. Environmental factors deserve more attention than they have gotten to date, because they have a large potential impact on obesity on a population level [5].

Several theoretical frameworks containing environmental influences on physical activity have been designed for the fields of health promotion [6,7] and policy research [8]. A framework often referred to is the ANalysis Grid for Environments Linked to Obesity (ANGELO) [9], which relates the environment to energy intake/dietary habits (not included in this study) and energy expenditure/physical activity. The ANGELO distinguishes four categories of environmental factors (physical, economic, political and socio-cultural) which may operate at the micro-level (home, school or neighbourhood) as well as the macro-level (city or country) [9]. Because different policies can influence the macro-level and the micro-level, governing bodies should take both levels into consideration for policy-making in an expanded view [8].

Two review studies summarized existing literature by using the ANGELO framework, one of which focused specifically on youth, both assessed dietary habits and PA [10,11]. The shared conclusion was that for PA, the micro-level has been relatively well-studied, whereas the macro-level has been virtually ignored. At the time of these reviews, in 2009, only one study could be identified that assessed the macro-level environment in relation to obesity [12].

One more recent study analysed macro-level variables in relation to leisure time physical activity (LTPA) in Europe [13]. Higher Gross domestic product (GDP), fat (food) availability and urbanization (urban population, total and new passenger cars) were all associated with higher levels of LTPA; political factors were not associated with LTPA. Another recent study, studied the association of the Human Development Index (HDI) with total level of PA across the world [14]. This study concluded that in countries with a higher HDI a larger percentage of the population did not meet PA recommendations.

The main difference between these studies is the outcome measure; whereas LTPA was studied by van Tuyckom [13], Dumith et al studied PA [14]. LTPA levels are generally higher among higher occupational classes [15]. Similarly, LTPA levels may contribute more to overall PA in more developed countries, while occupational PA, household PA and active transport may contribute more to overall PA levels in lower income countries.

To summarise, current evidence on links between the macro-level environment and PA is limited and mostly comes from comparisons between high-income countries. This exploratory study aims to add to the literature by assessing for a broad range of factors at the country-level whether they are associated with levels of overall PA in low- and middle-income countries. This study used data from the World Health Survey (WHS), which included 287,250 respondents from 72 countries [16]. A notable strength of the WHS is that it contains comparable data from many countries around the World, including many low- and middle-income countries. These data are combined with indicators of the environment on a macro-level.

## Methods

For this study data from the World Health Survey (WHS) of the World Health Organization (WHO) were analyzed in June 2011. This international, cross-sectional interview survey was implemented between 2002 and 2005 in 72 countries [17]. More details about the WHS can be found online [16]. Two countries chose not to release results and 19 countries were excluded because they did not implement the module which contained the short form of the International Physical Activity Questionnaire-Short Form (IPAQ-S). An additional 11 countries were excluded because the percentage of missing values for the three types of PA (see below) was over 15%. Another country was excluded because no data on occupational class was available. Lastly one country was excluded because no data on gender equality (as described below) could be obtained. Furthermore, 188 individuals from various countries with missing values on one or more covariates were excluded. Because the IPAQ-S has only been validated for respondents aged 18 to 69 years those younger than 18 or older than 69 years of age were excluded. This resulted in a final population of 38 countries with a total of 177,035 individual respondents. Additional data on number of respondents per country can be found in Table 1.

According to the Medical Ethics Review Committee of the AMC, the Dutch Medical Research Involving Human Subjects Act (WMO) does not apply to this study and therefore no official approval was required. PA was measured using the IPAQ-S, a validated tool designed to assess PA behaviours across all fields. PA is measured as the total of all types of PA (leisure-time, occupation, household and transport) [18]. It assessed vigorous-intensity PA, moderate-intensity PA and walking, separately. In the IPAQ-S, respondents were asked to report the frequency (number of days per week on which at least 10 minutes of PA was performed) and duration (in hours and minutes per day) of PA. Only the measures of frequency of PA were used in this study, because data on the duration of activity contained too many missing values (>40% overall). With these frequency measures, respondents were classified into ‘active’ and ‘inactive’ for all three categories. Respondents were classified as active if they participated in vigorous PA for three days or more per week, moderate PA for five days or more per week and walking for five days or more per week. These cut-off values are derived from the scoring form of the IPAQ-S [18]. To investigate whether results were robust to the choice of these cut-off values, sensitivity analysis was performed where higher and lower cut-off values were used. The results as reported below did not change significantly when using alternative cut-off values.

**Table 1:** Descriptive information of study population, stratified by country

**Country**	**N**	**Mean age**	**% Male**	**% High occupation**^a^	**% High education**^b^
		**In years**			
Bangladesh	5,168	36.8	46.1	15.8	8.6
Brazil	4,626	39.0	43.6	22.8	29.5
Burkina Faso	4,430	34.3	46.8	11.1	2.9
China	3,637	42.3	49.3	23.3	29.0
Comoros	1,413	38.5	45.3	10.1	5.7
Cote d’Ivoire	2,584	34.0	57.2	16.4	13.0
Croatia	783	46.9	41.9	29.6	17.0
Czech Republic	776	42.5	44.9	37.0	49.7
Dominican Republic	4,121	38.5	46.2	21.8	5.1
Estonia	847	45.1	36.3	39.1	79.5
Georgia	2,128	42.8	44.4	22.2	94.5
Ghana	3,232	37.9	46.0	19.9	4.4
Guatemala	3,693	37.5	38.3	14.6	12.1
Hungary	1,115	44.4	42.1	30.2	68.9
India	8,317	37.0	48.4	12.6	21.9
Kazakhstan	4,305	40.3	34.4	58.0	96.8
Lao PDR	4,618	36.7	46.7	9.7	9.9
Malawi	4,810	33.4	42.3	12.0	1.2
Malaysia	5,638	39.4	44.4	27.2	44.3
Mauritania	2,733	36.6	37.1	9.9	9.8
Mauritius	3,605	39.7	48.7	22.7	13.6
Mexico	35,045	38.0	42.7	18.7	24.6
Myanmar	5,540	38.9	43.5	7.9	9.8
Namibia	3,507	35.3	40.2	17.9	4.7
Nepal	7,631	36.8	42.0	5.9	5.2
Pakistan	5,818	35.3	55.4	16.3	15.0
Paraguay	4,776	37.3	45.7	23.1	12.1
Philippines	9,591	37.5	46.3	15.4	16.6
Russian Federation	3,410	44.7	38.1	47.7	71.4
South Africa	2,172	36.4	47.4	22.3	35.0
Spain	4,846	45.4	41.9	26.7	37.7
Sri Lanka	4,956	38.9	47.2	15.6	23.0
Tunisia	4,411	38.0	45.9	15.5	30.4
Ukraine	1,899	42.5	37.4	41.3	93.5
United Arab Emirates	1,111	36.4	52.4	41.3	66.1
Uruguay	2,626	41.9	49.9	45.5	32.9
Zambia	3,598	34.4	45.1	9.8	5.5
Zimbabwe	3,519	35.1	36.7	8.8	5.1
TOTAL	177,035	38.1	44.4	19.4	23.7

^a^ White collar worker (e.g. legislator, clerk or service worker).

^b^ University/Post graduate/High school completed.

The covariates used on the micro-level were: sex, age, occupational class and education. Age was categorized into mostly five-year age categories (18-20, 21-25…61-65, 66-69). Occupational class distinguished five types of employment: white collar jobs (e.g. legislator, clerk or sales worker), blue collar jobs (e.g. craft trades worker, elementary worker or plant operator), agricultural or fishery jobs, armed forces and not working for pay. The education of respondents was considered ‘high’ if they had completed high school, university or post-graduate school, while the other categories were secondary school, primary school, less than primary school and no formal education. Of the respondents, 44.4% was male with a mean age of 38.1 (standard deviation = 13.6 years). Further details are given in Table 1.

The environmental characteristics to be included in this study were selected on the basis of three criteria: (A) each of the four categories from the ANGELO framework had to be represented; (B) within these categories, variables had to measure distinct dimensions of this environmental factor; (C) internationally comparable data had to be available for most of the WHS countries. The following variables were selected:

Average yearly temperature was defined as the mean of average maximum and minimum temperature (in degrees Celsius) per month in the capital city. Data were obtained from climatetemp in March 2011 [19].

Motor vehicle density was measured as the number of cars, trucks, and buses per 1,000 people. Data were from 2002-2008 and were taken from the World Bank Database for most countries [20]. For Cote d’Ivoire and Mauritania, data were from the CIA World Factbook [21].

Rural population was measured as the percentage of people living in rural areas. Data from 2002 were obtained from the World Bank estimates [20].

Economic development was measured as the gross domestic product (GDP) (in 2011 U.S. dollars) divided by midyear population. Data on GDP per capita were retrieved from the World Bank for 2002 [20], with the exception of Myanmar (2007) [22].

The average governance indicator was measured as the mean score of governance as judged by survey respondents; based on six categories: control of corruption, government effectiveness, political stability and absence of violence/terrorism, rule of law, regulatory quality and voice and accountability. These data were taken from World Bank Policy Research from 2002 [20].

Literacy rate was measured as the percentage of adults who can read and write a short, simple statement on their everyday life. Data were from 2000-2008, obtained from the United Nations Educational, Scientific and Cultural Organisation (UNESCO) [23].

The Social Institutions & Gender Index (SIGI) was measured as a composite indicator of gender equality. The SIGI data of 2009 were obtained from the OECD Gender, Institutions and Development database [24]. However, SIGI values were not available for seven countries (Comoros, Czech Republic, Estonia, Hungary, Malaysia, Mexico and Spain). The relative positions of these countries on another gender inequality measure, the Gender Development Index (GDI) were used to estimate SIGI values for those countries. The GDI data were taken from the United Nations Development Programme’s Human Development Report [25].

The scales and percentile distributions of these macro-level variables can be found in Table 2.

**Table 2:** **Sources and percentile distribution of country-level characteristics**^**a**^

**Environmental indicators**	**Source**	**Unit**	**P10**	**P25**	**P50**	**P75**	**P90**
Physical environment							
Average Yearly Temperature	CT^b^	° Celsius	8.57	14.08	20.15	26.03	27.00
Motor Vehicle Density	WB^c^, CIA	Ln(Motor vehicles per 1000 people)	1.94	2.65	4.47	5.15	5.75
Rural Population	WB^c^	0 – 20	4.86	7.00	10.14	13.28	15.58
Economic environment							
GDP, per capita	WB^c^, EIU	Ln( Current US $)	5.67	6.12	7.22	8.20	8.78
Political environment							
Average Governance Indicator	WB^c^	0 – 5	1.47	1.77	2.27	2.79	3.27
Socio-cultural Environment							
Literacy Rate	UIS^d^	0 – 20	9.91	13.82	17.77	19.40	19.89
Social Institutions & Gender Index (SIGI)	OECD, HDR^e^	0 – 10	7.73	8.52	9.55	9.81	9.94

^a^ P10 = 10^th^ percentile etc.

^b^ CT = Climatetemp.

^c^ WB = World Bank.

^d^ UIS = UNESCO Institute for Statistics.

^e^ HDR = United Nations Development Programme’s Human Development Reports.

### Statistical analyses

The statistical package Stata (version 11.1) was used for all analyses. Age-standardized prevalence rates of the three types of PA were calculated by means of the direct method, using the WHO world standard [26]. Prevalence rates were calculated per country and sex. To assess the relationships between characteristics of countries and individual PA, multilevel logistic modelling (also called hierarchical modelling) was used. This technique takes into account the dependency of observations within a cluster (in this case a country) [27]. It controls for confounding on the micro-level, like regular regression analysis, but in addition controls for confounding at the macro-level. To first assess the micro-level associations, a model was used which included age, occupation and education as independent variables; this was modelled for each of the three types of PA separately. To assess associations with macro-level variables, three nested models were made; the first model only controlled for age, the second model added occupation and education, and based on the results presented below, the third model also controlled for temperature and GDP on the macro-level. All macro-level variables were analyzed independently with the control variables included. When controlling for GDP, an interaction variable to control for interaction between GDP and education was also included (not where GDP was included as determinant). All analyses were stratified by sex, so this was not included as a covariate.

For the following variables the scales have been adjusted to make interpretation of the OR’s more comparable or meaningful. Both rural population and literacy rate were percentages which were then divided by five to create scales from 0 to 20. For the average governance indicator, a positive scale was made so that it ranges from 0 to 5 instead of from -2.5 to 2.5, where 5 indicated the ‘best’ possible governance. The SIGI has been inverted so that a higher value now indicates higher gender equality, and the scale has been multiplied by 10 so it ranges from to 0 to 10. Finally, in order to account for non-linear relationships with PA, the natural logarithm of the motor vehicle density and GDP were used.

To assess the magnitude of between-country variation as compared to within-country variation, the amount of total variation in PA that is due to variation on the macro-level was calculated with the measure Rho.

## Results

**Figure 1:**
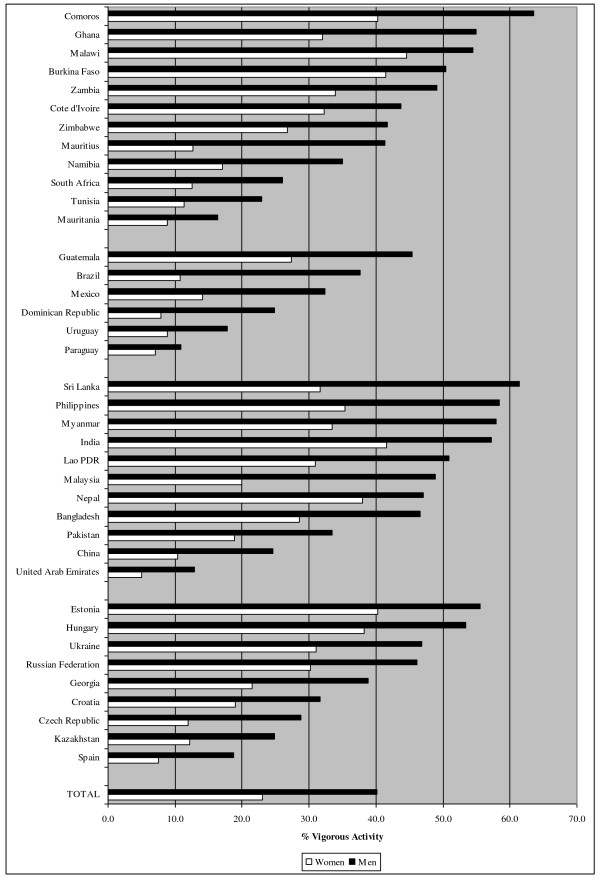
Prevalence of vigorous physical activity stratified by country.

**Figure 2:**
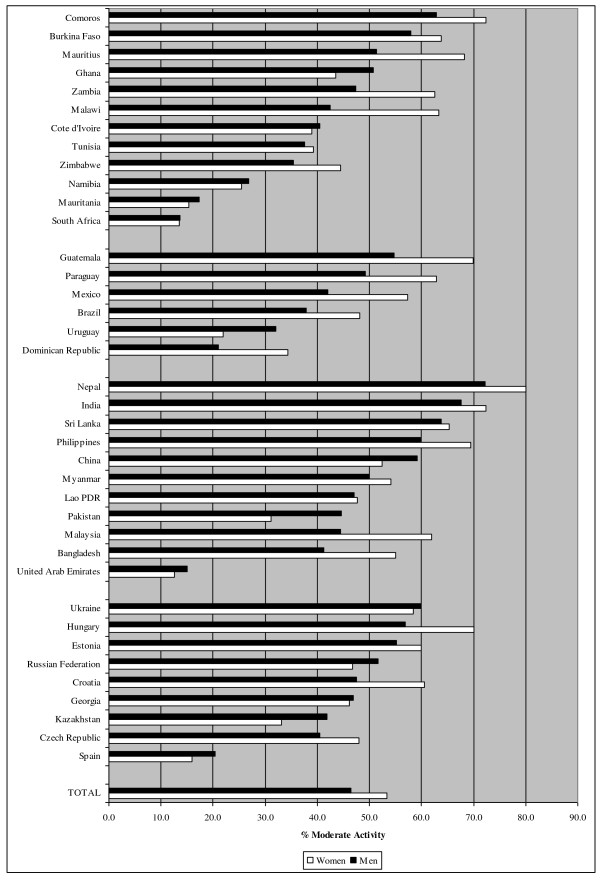
Prevalence of moderate physical activity stratified by country.

**Figure 3:**
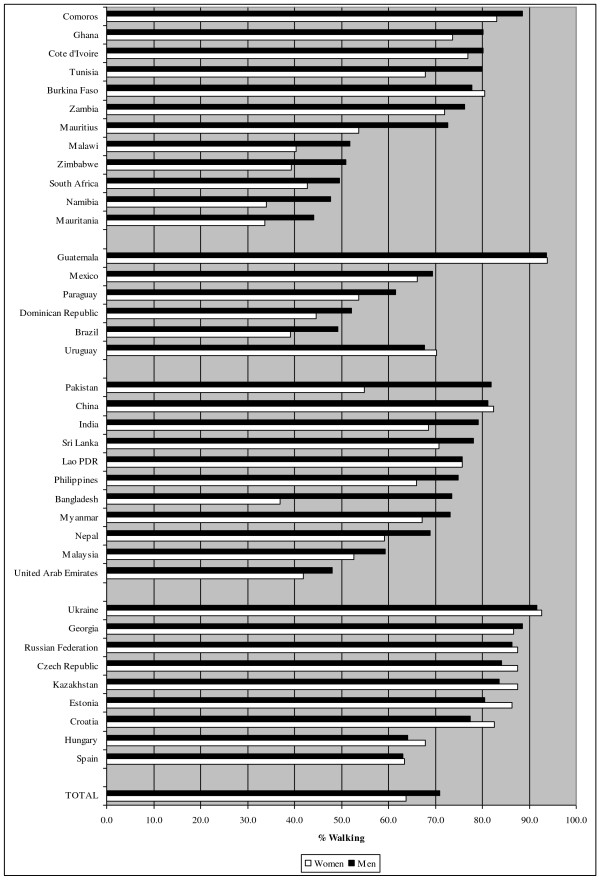
Prevalence of walking stratified by country.

Figures 1, 2 and 3 show the prevalence of PA, defined as the percentage of the population that is considered active, for vigorous PA, moderate PA and walking. In total, 30.8%, 47.5% and 68.0% of respondents were classified as active in vigorous-intensity PA, moderate-intensity PA and walking respectively. However, the figures varied strongly between countries (e.g. 10.9%-63.5% for vigorous PA in men, 5.0%-44.5% for vigorous PA in women). Residents of African and Southeast-Asian countries were on average more active in vigorous PA, compared to European and South-American countries. For moderate PA and walking no clear pattern was observed across regions.

All included micro-level characteristics were found to be associated with PA (Table 3). Lower PA levels were observed with increasing age and this effect was strongest in vigorous PA among men aged 66-69 (OR = 0.31, 95% CI: 0.28-0.35). Higher levels of activity were seen among those working in agriculture or blue collar jobs when compared to white collar jobs, where the effect was also the strongest for male in vigorous PA (OR = 3.00, 95% CI: 2.85-3.15 and OR = 1.84, 95% CI: 1.76-1.93 respectively). Lower education levels were associated with higher activity levels, with uneducated men having the highest levels of vigorous PA (OR = 1.79, 95% CI: 1.67-1.91).

**Table 3:** Associations between individual-level characteristics and physical activity stratified by sex

	**Men**							**Women**						
	**N**	**OR 95% CI**						**N**	**OR 95% CI**					
		**Vigorous**		**Moderate**		**Walking**			**Vigorous**		**Moderate**		**Walking**	
Occupation														
White collar^a^	18,099	1	-	1	-	1	-	16,158	1	-	1	-	1	-
Blue collar^b^	21,845	1.84	1.76-1.93	1.17	1.12-1.23	0.96	0.92-1.01	10,535	1.37	1.28-1.47	1.26	1.19-1.33	0.98	0.92-1.04
Agriculture	21,198	3.00	2.85-3.15	1.30	1.24-1.36	1.26	1.19-1.33	10,748	2.71	2.53-2.90	1.46	1.37-1.56	1.17	1.09-1.25
Armed forces^c^	1,172	1.39	1.22-1.58	1.09	0.96-1.24	1.11	0.97-1.27							
Not working for pay	16,282	1.01	0.96-1.07	0.77	0.73-0.81	0.85	0.80-0.89	60,849	0.97	0.92-1.03	1.10	1.06-1.15	0.74	0.71-0.78
Education														
Higher education^d^	19,686	1	-	1	-	1	-	22,266	1	-	1	-	1	-
Secondary school	21,497	1.15	1.10-1.21	1.09	1.04-1.14	1.01	0.96-1.06	25,042	0.95	0.90-1.00	1.23	1.18-1.29	1.02	0.98-1.07
Primary school	16,890	1.42	1.35-1.50	1.03	0.98-1.08	0.97	0.92-1.02	19,042	1.20	1.13-1.28	1.24	1.18-1.30	1.00	0.95-1.05
Less than primary	9,883	1.62	1.51-1.73	0.98	0.92-1.04	0.98	0.93-1.04	11,990	1.43	1.34-1.54	1.20	1.13-1.27	0.99	0.93-1.05
None	10,640	1.79	1.67-1.91	1.08	1.02-1.15	0.91	0.85-0.98	20,099	1.68	1.57-1.80	1.19	1.12-1.26	1.03	0.97-1.09

^a^ White collar (e.g. legislator, clerk or sales worker).

^b^ Blue collar (e.g. craft trades worker, elementary worker or plant operator).

^c^ The group of women working in the armed forces was too small to include in these results.

^d^ Post graduate, university or high school.

Associations between macro-level environment and PA are given in Table 4. Controlling only for age only (Model 0) average yearly temperature, motor vehicle density, rural population, GDP, average governance indicator and literacy rate were associated with statistical significance to at least one of the PA outcomes. For example, the OR of 0.78 implied that the odds for a man to be vigorously active are 22% lower for a man in a country with a high compared to a lower GDP.

**Table 4:** Associations between country-level environmental characteristics and physical activity, stratified by sex

**Environmental factor**	**Type of activity**	**OR 95% CI**											
		**Men**						**Women**					
		**Model 0**		**Model 1**		**Model 2**		**Model 0**		**Model 1**		**Model 2**	
Temperature	Vigorous	1.01	0.98-1.04	0.98	0.95-1.01	0.97	0.94-1.00	1.01	0.97-1.04	0.98	0.95-1.02	0.96	0.93-0.99
	Moderate	0.99	0.97-1.02	0.99	0.96-1.02	0.97	0.94-0.99	1.00	0.97-1.04	1.00	0.96-1.03	0.98	0.94-1.01
	Walking	0.97	0.94-1.00	0.97	0.94-1.00	0.95	0.92-0.98	0.93	0.90-0.97	0.94	0.90-0.97	0.92	0.89-0.96
Motor vehicle density	Vigorous	0.88	0.76-1.02	1.02	0.87-1.19	†	†	0.82	0.70-0.95	0.94	0.80-1.10	1.28	0.95-1.72
	Moderate	0.89	0.78-1.02	0.92	0.80-1.05	1.15	0.89-1.50	0.90	0.75-1.07	0.93	0.78-1.11	1.28	0.88-1.85
	Walking	0.98	0.84-1.15	0.99	0.85-1.16	1.17	0.87-1.58	1.15	0.95-1.40	1.16	0.96-1.40	1.45	1.03-2.04
Rural population	Vigorous	1.08	1.03-1.13	1.03	0.98-1.09	1.05	0.97-1.15	1.11	1.05-1.16	1.06	1.00-1.11	1.06	0.97-1.14
	Moderate	1.06	1.02-1.11	1.05	1.01-1.10	1.05	0.98-1.12	1.08	1.02-1.14	1.06	1.00-1.13	1.07	0.97-1.18
	Walking	1.01	0.96-1.07	1.01	0.95-1.07	0.99	0.91-1.07	0.97	0.90-1.04	0.97	0.90-1.04	0.98	0.89-1.07
GDP	Vigorous	0.78	0.67-0.92	0.93	0.78-1.11	0.85	0.70-1.04	0.70	0.60-0.83	0.84	0.70-1.00	0.76	0.63-0.91
	Moderate	0.80	0.69-0.94	0.83	0.71-0.97	0.76	0.65-0.89	0.80	0.65-0.98	0.84	0.68-1.03	0.79	0.63-0.98
	Walking	0.89	0.74-1.07	0.91	0.76-1.09	0.79	0.66-0.94	1.01	0.80-1.28	1.03	0.82-1.30	0.84	0.68-1.03
Governance indicator	Vigorous	0.79	0.59-1.08	0.99	0.72-1.36	1.21	0.77-1.92	0.69	0.49-0.95	0.85	0.61-1.16	1.16	0.75-1.80
	Moderate	0.76	0.57-1.01	0.79	0.60-1.04	1.08	0.74-1.57	0.77	0.53-1.11	0.81	0.56-1.16	1.07	0.63-1.84
	Walking	0.77	0.56-1.06	0.80	0.58-1.09	0.84	0.55-1.29	0.91	0.61-1.38	0.93	0.62-1.40	0.83	0.50-1.39
Literacy rate	Vigorous	0.97	0.91-1.02	1.02	0.97-1.09	†	†	0.94	0.88-0.99	0.99	0.93-1.05	1.02	0.94-1.09
	Moderate	0.98	0.93-1.03	0.99	0.94-1.04	1.02	0.96-1.09	0.98	0.91-1.05	0.99	0.93-1.06	1.04	0.95-1.14
	Walking	0.99	0.94-1.05	1.00	0.94-1.06	0.98	0.91-1.05	1.05	0.97-1.13	1.05	0.97-1.13	0.99	0.91-1.09
SIGI	Vigorous	0.96	0.75-1.22	1.08	0.85-1.39	1.10	0.85-1.44	0.86	0.66-1.12	0.99	0.77-1.27	1.05	0.81-1.35
	Moderate	0.99	0.79-1.25	1.01	0.81-1.27	1.07	0.86-1.33	1.04	0.77-1.39	1.08	0.81-1.44	1.20	0.88-1.62
	Walking	1.10	0.85-1.41	1.11	0.86-1.42	1.09	0.85-1.39	1.39	1.02-1.88	1.38	1.02-1.85	1.25	0.94-1.66

Model 0 corrects only for age, Model 1 corrects for age, educational attainment and occupational class and Model 2 corrects for age, educational attainment and occupational class on the individual level and average yearly temperature and GDP on the country-level (except in the model where these were included as determinant).

†: Model could not be fitted, due to lack of convergence.

After correcting for occupation and education (Model 1) the associations of average governance indicator and literacy rate remained mostly unchanged, but were no longer statistically significant. In contrast, a greater proportion of women were found to walk in countries with higher gender equality (OR = 1.38, 95% CI: 1.02-1.85). The factors that were significantly associated with PA after controlling for micro-level and macro-level factors (Model 2) were average yearly temperature (OR = 0.92, 95% CI: 0.89-0.96), motor vehicle density (OR = 1.44, 95% CI: 1.02-2.02) and GDP (OR = 0.76, 95% CI: 0.65-0.89).

Associations controlled for individual and macro-level factors (Model 2) were also stratified by occupational class (Table 5). Here the same factors were found to be associated with PA. The effects were most pronounced in both the white collar group and those not working for pay. In the association between GDP and moderate PA OR’s were 0.81 (95% CI: 0.69-0.96) for the white collar group and 0.82 (95% CI: 0.67-1.00) for those not working for pay compared to 0.87 (95% CI: 0.72-1.04) for the blue collar group and 0.91 (95% CI: 0.76-1.09) for agricultural workers.

**Table 5:** **Associations between country-level environmental characteristics and physical activity, stratified by occupational class**^**a**^

**Environmental factor**	**Type of activity**	**OR 95% CI**							
		**White Collar**^**b**^		**Blue Collar**^**c**^		**Agriculture**		**Not Working**	
Temperature	Vigorous	0.97	0.94-0.99	0.96	0.93-1.00	0.99	0.94-1.03	0.96	0.93-1.00
	Moderate	0.98	0.95-1.01	0.97	0.93-1.00	0.96	0.93-0.99	0.97	0.94-1.01
	Walking	0.93	0.90-0.96	0.93	0.90-0.97	0.94	0.90-0.98	0.94	0.90-0.97
Motor vehicle density	Vigorous	1.33	1.03-1.71	1.37	0.97-1.92	1.26	0.83-1.92	1.46	1.09-1.97
	Moderate	1.20	0.89-1.62	1.15	0.84-1.59	1.10	0.81-1.48	1.36	0.96-1.94
	Walking	1.24	0.91-1.69	1.27	0.93-1.73	1.21	0.85-1.73	1.43	1.02-2.00
Rural population	Vigorous	1.02	0.95-1.10	1.06	0.96-1.16	1.06	0.95-1.19	1.06	0.97-1.15
	Moderate	1.07	0.99-1.16	1.08	1.00-1.18	1.04	0.96-1.13	1.05	0.95-1.15
	Walking	0.98	0.90-1.07	0.99	0.91-1.08	0.99	0.89-1.09	0.98	0.89-1.08
GDP	Vigorous	0.96	0.82-1.12	0.92	0.76-1.12	0.89	0.70-1.14	0.81	0.68-0.98
	Moderate	0.81	0.69-0.96	0.87	0.72-1.04	0.91	0.76-1.09	0.82	0.67-1.00
	Walking	0.92	0.75-1.12	0,90	0.74-1.10	0.98	0.79-1.22	0.98	0.79-1.21
Governance indicator	Vigorous	1.31	0.90-1.91	1.16	0.70-1.91	1.26	0.68-2.34	1.11	0.70-1.24
	Moderate	1.04	0.67-1.60	0.94	0.59-1.49	1.03	0.66-1.61	1.07	0.64-1.80
	Walking	0.77	0.50-1.20	0.81	0.51-1.27	0.77	0.46-1.29	0.88	0.53-1.47
Literacy rate	Vigorous	1.03	0.96-1.10	1.06	0.97-1.15	1.06	0.95-1.17	1.02	0.94-1.10
	Moderate	1.03	0.96-1.11	1.03	0.95-1.12	1.02	0.95-1.10	1.04	0.95-1.14
	Walking	0.99	0.91-1.07	0.99	0.91-1.07	0.99	0.90-1.08	0.98	0.90-1.07
SIGI	Vigorous	1.12	0.90-1.39	1.18	0.89-1.57	1.09	0.76-1.57	1.10	0.85-1.43
	Moderate	1.08	0.84-1.38	1.05	0.81-1.37	1.03	0.79-1.34	1.28	0.96-1.71
	Walking	1.10	0.85-1.42	1.12	0.86-1.45	1.12	0.82-1.51	1.23	0.93-1.63

^a^ All models are corrected for age, educational attainment and occupational class on the individual level and average yearly temperature and GDP on the country-level (except in the model where these were included as determinant).

^b^ White collar (e.g. legislator, clerk or sales worker).

^c^ Blue collar (e.g. craft trades worker, elementary worker or plant operator).

The mean Rho of the first analyses (Model 0) was 0.14 with a 95% CI of 0.10-0.21, indicating that 14% of the total variation in PA between all individuals is explained by variation between countries. When correcting for education and occupation in Model 1 these values remained unchanged, but when correcting for additional factors in Model 2 the Rho decreased to 0.12 (95% CI: 0.08-0.17). When looking at types of PA separately the Rho values are 0.11 (95% CI: 0.07-0.16), 0.12 (95% CI: 0.08-0.17) and 0.12 (95% CI: 0.08-0.18) for vigorous PA, moderate PA and walking respectively.

## Discussion

The percentage of respondents classified as active varied widely between countries. Moreover cross-national patterns varied for the three types of PA. A few statistically significant relationships between PA and macro-level characteristics were found. Levels of PA were highest in countries with a relatively low GDP. A higher motor vehicle density and higher gender equality were associated with more walking for women. Furthermore, a higher temperature was associated with less PA in general and a lower percentage of rural population was associated with less vigorous and moderate PA. Governmental functioning and literacy rate were not found to be associated with any type of PA.

### Limitations

One limitation of this study is the fact that it was not possible to incorporate knowledge about the exact duration of PA in the analyses, because the amount of missing values on these measures was considered too high (57.1% of respondents had missing values for both hours and minutes spent in vigorous PA). Since it is plausible that some of the countries whose citizens are relatively active in terms of frequency would be less active in terms of duration, the results might have been different if data on duration of PA had been available.

A notable strength of this study is the availability of data on PA across many countries, collected with a single methodology. In this approach the WHO has set up rigorous procedures to minimize the differences in interpretation [28]. This is needed because the concept of PA and interpretations of intensity are likely to vary among people from different countries and cultures. Despite the rigorous techniques implemented by the WHO, issues of comparability cannot be underestimated.

PA-questionnaires like the IPAQ-S have been reported to be prone to overestimating PA [29-31]. However it is hard to ascertain whether this effect varies between countries. Another limitation of the IPAQ-S is that, while it separates walking, moderate and vigorous PA, it fails to distinguish domains of PA such as occupational, leisure-time, and transport. The contributions these domains make to overall PA can vary widely; therefore an overall PA measure cannot reflect the possibly much larger variations between countries in these specific domains. Furthermore, a review of studies that compared self-reported PA to directly measured PA (e.g. accelerometers) concludes that self-reported measures generally are unreliable [32]. Future research in the field of PA could be of significant added value by using more reliable tools, such as accelerometers to measure PA objectively [33].

Countries with over 15% of missing values on the PA measures were excluded. Data for remaining countries still had some missing values (mean 4.7%). The percentage of item-non response did not show correlations across countries with determinants or with the prevalence of PA. This suggests that this problem may not have influenced the results to a significant degree.

The total response rates per country were said to be available from the WHS website, but could not be found there [16]. Other studies that used the WHS did report response rates [34-36]. One study reported overall response rates over 70% in all countries except for the Czech Republic (23.9%) with individual level response rates varying between 82.2 and 100% [35]. Another study reported that response rates were over 80 percent for all regions [36]. From this information, it is not possible to ascertain whether response was selective to PA, and how much it could have influenced our results.

### Interpretation of results

This is one of the first studies to assess associations between macro-level environmental factors and PA. One other study, on international variations in LTPA across Europe, observed patterns of associations quite different from those found in this study [13]. That study found a higher GDP to be associated with more LTPA, while this study found a higher GDP to be associated with less PA in general. A higher rural population, higher number of passenger cars and higher government effectiveness were also associated with more LTPA in the other study. That study compared only European countries, which in terms of economic development are quite different from most of the countries included in this study. Also, the fact that the other study used LTPA as outcome makes it hard to compare the results of the two studies, as it is known that patterns of PA and LTPA differ widely. Generally speaking, in developing countries people are more active in occupational settings and transport, while in developed countries people are more active in their leisure time [37].

One of the physical characteristics of the environment which was associated with PA was temperature. People in warmer countries were found to be less likely to be active. This in itself is not surprising and is in agreement to findings of studies assessing seasonal variations within countries [38-40]. It seems plausible that the effect of temperature would differ per climate region. In countries with a relatively cold climate an increase in temperature could cause more people to be active, whereas in a country with a relatively warm climate, a further increase in temperature might cause people to be less active. In statistical terms, the association between temperature and PA could be expected to be non-linear. Therefore a normal function was compared with a squared and a cubic function. However, no evidence for a non-linear association was found within this set of countries.

Respondents from countries with a higher GDP (more developed countries) were less likely to be physically active. This corresponds to the findings of earlier research, although patterns for general PA and LTPA seem to be in the opposite direction [14,37,41,42]. It has mostly been attributed to the modern ‘Western’ lifestyle, which discourages people to be active. This lifestyle is facilitated by mechanized labour, sedentary occupations and motorized transport [43].

A higher motor vehicle density appeared to be related to more PA. This stands in contrast to earlier research that has shown trends of decreasing walking and cycling while numbers of motor vehicles increase [44,45]. One possible explanation for our finding is that this association reflects the result of a third underlying factor such as the extent of geographical mobility in a country. For example, if a society has higher need for mobility in general this could be reflected in increased levels of both motorized and physically active transport. Research in Europe also showed a higher passenger car density to be associated with more LTPA [13].

A higher percentage of the population living in rural areas was found to be associated to higher levels of PA and this effect was most pronounced in those working in non-agricultural occupations. Other studies at sub-national level reported urban dwellers to be less active compared to those living in rural areas in the same country [34,46-48]. However, these effects are not fully equivalent to those found in this study, as the urbanization of a country is indicative of the geography and development of a country (which we controlled for by adding GDP to the model), while individual place of residence depends upon other factors.

Although not significant in the full model, the average governance indicator and literacy rate showed associations with PA. A higher governance indicator was associated with less PA; possibly this is an effect of the general state of development of a country [14]. A higher literacy rate was also associated with less PA, which is similar to the effect also found in this study, that a higher education was associated with lower levels of PA. This effect remained after controlling for education.

The positive effect of high gender equality (SIGI) on PA was most obvious for walking among women. This can perhaps be explained by the large differences in the degree of women’s freedom between countries with high and low gender equality. These differences are determined by a combination of economic, societal and religious factors [49].

There were some pairs of countries (Bangladesh and Pakistan, Croatia and Hungary) that were fairly similar with regard to all included macro-level environmental variables, but that nonetheless had quite different PA prevalence rates. In-depth comparisons of such countries, using information on specific domains of PA, are needed to reveal the specific environmental factors that have caused differences in total PA.

## Conclusions

This study, one of the first to assess the relationship between the macro-level environment and PA in middle- and low-income countries, suggests that several aspects of the environment on the macro level, as described in the ANGELO-framework, may have an impact on PA. Though the environmental factors might not be easily influenced by policy, the outcomes of this study can be used to predict future changes in the prevalence of PA at national levels. The observed relationships suggest that country-wide changes in the environment and society may result in large scale changes in PA prevalence. Low- and middle-income countries with strong economic growth and urbanization can be seen as at risk of developing reduced levels of PA. This transition in PA is likely to have large negative public health effects; therefore preventive strategies should be developed and put into practice.

## Abbreviations

95% CI: 95% Confidence Interval; ANGELO: Analysis Grid for Environments Linked to Obesity; CIA: Central Intelligence Agency; EIU: Economist Intelligence Unit; GDI: Gender Development Index; GDP: Gross Domestic Product; HDI: Human Development Index; IPAQ-S: International Physical Activity Questionnaire – Short Form; LTPA: Leisure Time Physical Activity; OECD: Organisation for Economic Co-operation and Development; OR: Odds Ratio; PA: Physical Activity; SIGI: Social Institutions & Gender Index; UNESCO: United Nations Educational, Scientific and Cultural Organisation; WHO: World Health Organization; WHS: World Health Survey.

## Competing interests

The authors declare that they have no competing interests.

## Authors’ contributions

JB and AK conceived the article, JB analysed and interpreted the data and led the writing. MW prepared and analysed the data and provided critical revisions. TV helped to interpret the data and provided critical revisions. AK co-authored the article and provided critical revisions. All authors read and agreed with the final version of the paper.

## Authors’ information

All authors except T.Visscher are with the Department of Public Health, Academic Medical Centre, University of Amsterdam. T. Visscher is with the Research Centre for the Prevention of Overweight, VU University Amsterdam/Windesheim University of Applied Sciences, Zwolle.
